# Supplementary material for: Herpes simplex encephalitis due to a mutation in an E3 ubiquitin ligase
Source: Nat Commun. 2024 May 10;15:3969. doi: 10.1038/s41467-024-48287-0 (PMC11087577; doi:10.1038/s41467-024-48287-0)

Inventory of supplementary information

Supplementary Tables

Supplementary Table 1. Identification of a heterozygous *WWP2* missense mutation

Supplementary Table 2. Experimental structures fetched and used for the ΔΔG calculations.

Supplementary Table 3. Alignment metrics

Supplementary Table 4. AP-1 and NF-kB downstream enrichment for the difference in Poly(I:C) stimulation between WWP2 p.R841H and WWP2 p.R841.

Supplementary Table 5. Primers.

Supplementary Figures

Supplementary Figure 1. Conservation of the Arginine 841 in vertebrate WWP2 orthologues

Supplementary Figure 2. Conservation of the Arginine 841 in 38 WWP2 paralogues

Supplementary Figure 3. Maximal and Minimal Energetic impact observed by the introduction of a R814H mutation in the HECT domain of WWP2.

Supplementary Figure 4. WWP2-dependent IFN responses in PBMCs.

Supplementary Figure 5 Significant downregulation of NFKB pathway downstream target genes in WWP2 p.R841H compared to WWP2 p.R841.

Supplementary Figure 6. Significant downregulation of AP-1 downstream target genes in WWP2 p.R841H compared to WWP2 p.R841.

Supplementary Table 1. Identification of a heterozygous WWP2 missense mutation

Inheritance	Zygosity	Gene and attributes					Variants and attributes							Remarks
		Gene	DOMINO	KO mice (MGI)	Disease associated (OMIM)	Link with TLR3	Change	Frequency (gnomAD)	Origin	ClinVar	Amino-acid	MutScore	CADD	
Recessive	Homozygous	None												
	Compound heterozygous	*ZFYVE26*	*Not relevant for recessive inheritance*	No immunological phenotypes	Spastic paraplegia autosomal recessive	No	c.3881A>G	NA	Mother	No	Not conserved	0.053	15.9	Not retained: mother variant with low deleteriousness prediction scores; association with an another disease and no link with immunological phenotype or function.
							c.961A>C	0.0134%	Father	Yes (VUS)	Conserved	0.383	27.7	
		FLNB	*Not relevant for recessive inheritance*	Immune phenotype	Larsen syndrome, autosomal dominant; Atelosteogenesis, autosomal dominant	No	c.2237A>C	0.0012%	Mother	No	Conserved	0.800	26.5	Not retained: association with other unrelated diseases
							c.4186G>A	0.0028%	Father	No	Conserved	0.260	23.0	
							c.5327T>C	0.0028%	Father	Yes (VUS)	Conserved	0.864	28.4	
Dominant	*De novo*	*RAB13*	<0.9	No immunological phenotypes	NA	No	c.429T>G	0.0004%	*De novo*	No	Conserved	0.342	3.6	Not retained: DOMINO score lower than 0.9 suggesting the absence of an association with a dominant phenotype and no link with immunological phenotypes
		CNTNAP2	<0.9	No immunological phenotypes	NA	No	c.3427G>C	0.0032%	*De novo*	No	Conserved	NA	22.9	Not retained: DOMINO score lower than 0.9 suggesting the absence of an association with a dominant phenotype and no link with immunological phenotypes
	Heterozygous	*VANGL2*	0.994	No immunological phenotypes	Neural tube defects, autosomal dominant	No	c.931G>A	0.0018%	Mother	No	Conserved	0.609	31.0	Not retained: association with an another disease and no link with immunological phenotype or function.
		WWP2	0.987	Immune phenotype	NA	Yes	c.2522G>A	0.0012%	Mother	No	Conserved	0.933	30.0	Retained variant

VUS: variant of uncertain significance; red means attribute pointing towards benign; green means attribute pointing towards pathogenic.

Supplementary Table 2. Experimental structures fetched and used for the ΔΔG calculations.

Protein	PDB ID	Position of the Arg
ITCH	5c7m[11]	874
ITCH	4be8[12]	874
NEDD4	2xbb[13]	870
NEDD4	5c7j[11]	870
NEDD4	2xbf[13]	870
NEDD4	4bbn[12]	870
NEDD4	5c7j[11]	870
NEDD4	5c91[14]	870
NEDD4L	3jw0[15]	925
NEDD4L	3jvz[15]	925
NEDD4L	2oni	925
NEDD4L	5hpk[11]	925
NEDD4L	3tug	925
SMURF2	1zvd[16]	719
WWP1	5hpt[11]	893
WWP1	1nd7[17]	893
WWP1	5hps[11]	893
WWP1	6j1y[10]	893
WWP2	4y07[7]	841
WWP2	5tj7[9]	841
WWP2	5tj8[9]	841
WWP2	5tjq[9]	841
WWP2	6j1z[10]	841

Supplementary Table 3. Alignment metrics

METRIC	Medium					
	M1	M2	M3	M4	M5	M6
	WWP2 p.R841H			WWP2 p.R841		
TOTAL_READS	61404314	56700356	49370062	45803731	39504625	51486150
QUALITY_TRIMMED_BASES_CUTADAPT	3108195	2832236	2412693	2208621	1954858	2585471
DISCARDED_RRNA	1324677	1242723	1118972	874830	796844	961455
DISCARDED_LOW_COMPLEXITY	2015832	1904131	1562551	1420469	1182211	1519207
CLEAN_READS	58063805	53553502	46688539	43508432	37525570	49005488
CLEAN_READS_ALIGNED	57630213	53156202	46298847	43174694	37224466	48525756
PCT_CLEAN_READS_ALIGNED	99.25	99.26	99.17	99.23	99.20	99.02
CLEAN_ALIGNED_BASES	5714999190	5271242811	4584518715	4280282645	3684401479	4798146339
MEAN_READ_LENGTH	100.63	100.63	100.63	100.63	100.63	100.63
READS_ALIGNED_IN_PAIRS	0	0	0	0	0	0
PCT_READS_ALIGNED_IN_PAIRS	0	0	0	0	0	0
STRAND_BALANCE	0.49	0.49	0.49	0.49	0.49	0.49
UNMAPPED_READS	433592	397300	389692	333738	301104	479732
UNPAIRED_READ_DUPLICATES	45560639	41166756	36076528	33046874	28560896	37173925
READ_PAIR_OPTICAL_DUPLICATES	0	0	0	0	0	0
UNIQUELY_ALIGNED	53510871	49385004	42771873	39910933	34156643	45031471
MULTIPLE_ALIGNMENT	4119342	3771198	3526974	3263761	3067823	3494285
READ_ALIGNED_TO_REF_EXONS	49788822	45731048	39749318	37099139	31733428	41742606
NO_FEATURE	1720314	1824510	1347082	1239129	999908	1564898
AMBIGUOUS	2001735	1829446	1675473	1572665	1423307	1723967
TOO_LOW_AQUAL	0	0	0	0	0	0
NOT_ALIGNED	433592	397300	389692	333738	301104	479732
ALIGNMENT_NOT_UNIQUE	10518228	9685821	9010348	8362435	7952180	8953669
BIAS_INDEX_50	54.11	54.01	56.14	55.30	57.89	54.46
5OVER3_COV_RATIO	0.72	0.73	0.62	0.66	0.55	0.71
UNMAPPED_READS_ALIGNED_TO_MITOCHONDRIA	3659	3042	3911	3564	3849	3838
UNMAPPED_READS_ALIGNED_TO_MITOCHONDRIA_%	0.84	0.77	1.00	1.07	1.28	0.80
MAPPED_READS_ALIGNED_TO_MITOCHONDRIA	851676	732384	813191	819909	728887	631739
MAPPED_READS_ALIGNED_TO_MITOCHONDRIA_%	1.48	1.38	1.76	1.90	1.96	1.30

Supplementary Table 3 continued.

METRIC	3h Poly(I:C) stimulation					
	P1	P2	P3	P4	P5	P6
	WWP2 p.R841H			WWP2 p.R841		
TOTAL_READS	48728829	47270714	43680905	52993268	39831724	41632801
QUALITY_TRIMMED_BASES_CUTADAPT	2428027	2501289	2373526	2667698	2128498	2081268
DISCARDED_RRNA	1060753	1072967	1240241	1247690	885686	879336
DISCARDED_LOW_COMPLEXITY	1436123	1517397	1447652	1751882	1291267	1325801
CLEAN_READS	46231953	44680350	40993012	49993696	37654771	39427664
CLEAN_READS_ALIGNED	45783710	44182694	40427824	49563315	37319708	39121426
PCT_CLEAN_READS_ALIGNED	99.03	98.89	98.62	99.14	99.11	99.22
CLEAN_ALIGNED_BASES	4529804390	4375223110	4003958595	4909172403	3698618521	3880188262
MEAN_READ_LENGTH	100.62	100.59	100.62	100.63	100.62	100.63
READS_ALIGNED_IN_PAIRS	0	0	0	0	0	0
PCT_READS_ALIGNED_IN_PAIRS	0	0	0	0	0	0
STRAND_BALANCE	0.48	0.48	0.48	0.48	0.48	0.48
UNMAPPED_READS	448243	497656	565188	430381	335063	306238
UNPAIRED_READ_DUPLICATES	34448409	33172503	29731483	37897026	27149076	28731985
READ_PAIR_OPTICAL_DUPLICATES	0	0	0	0	0	0
UNIQUELY_ALIGNED	42499812	40931521	37526946	45977698	34615723	36306352
MULTIPLE_ALIGNMENT	3283898	3251173	2900878	3585617	2703985	2815074
READ_ALIGNED_TO_REF_EXONS	39041026	37738884	34506696	42144811	31806447	33421944
NO_FEATURE	1892503	1665129	1631048	2109850	1511784	1528196
AMBIGUOUS	1566283	1527508	1389202	1723037	1297492	1356212
TOO_LOW_AQUAL	0	0	0	0	0	0
NOT_ALIGNED	448243	497656	565188	430381	335063	306238
ALIGNMENT_NOT_UNIQUE	8358927	8273480	7343938	9114837	6871505	7136669
BIAS_INDEX_50	53.24	53.25	53.13	52.96	53.09	53.24
5OVER3_COV_RATIO	0.78	0.77	0.78	0.79	0.78	0.77
UNMAPPED_READS_ALIGNED_TO_MITOCHONDRIA	8744	8650	11398	7827	6745	4984
UNMAPPED_READS_ALIGNED_TO_MITOCHONDRIA_%	1.95	1.74	2.02	1.82	2.01	1.63
MAPPED_READS_ALIGNED_TO_MITOCHONDRIA	1631704	1616308	1507569	1776657	1474545	1319356
MAPPED_READS_ALIGNED_TO_MITOCHONDRIA_%	3.56	3.66	3.73	3.58	3.95	3.37

Supplementary Table 4. AP-1 and NF-kB downstream enrichment for the difference in Poly(I:C) stimulation between WWP2 p.R841H and WWP2 p.R841.

Pathway	FDR adjusted-p value	Normalized Enrichment Score
NF-kB atypical	4.02e-01	1.09
NF-kB non-canonical	4.02e-01	-1.10
NF-kB canonical	3.02e-02	-1.65
TNF: AP-1 Downstream	3.55e-05	-2.07

Complete pathways	FDR adjusted-p value	Normalized Enrichment Score
hsa:04064	7.32-02	-1.37
hsa:04668	7.89e-05	-1.90

P-values were computed using the adaptive multi-level split Monte-Carlo approach of R package fgsea. Positive and negative enrichment are considered (two-sided). P-values were corrected for multiple testing using False Discovery Rate. FDR stands for False Discovery Rate.

Supplementary Table 5. Primers.

	Forward	Reverse
TLR3	5’-GTGCCAGAAACTTCCCATGT-3’	5’-TCCAGCTGAACCTGAGTTCC-3’
WWP2	5’-CTTGTCGTGTCGTTCCGCTT-3’	5’-ACCATTTGCTTCACACTTGACTT-3’
TRIF	5’-GCCAGCAACTTGGAAATCAGC-3’	5’-GGGGTCGTCACAGAGCTTG-3’
IFNB	5’-CATTACCTGAAGGCCAAGGA-3’	5’-CAGCATCTGCTGGTTGAAGA-3’
ISG56	5’-CCTCCTTGGGTTCGTCTACA-3’	5’-GGCTGATATCTGGGTGCCTA-3’
MX1	5’-ACCTACAGCTGGCTCCTGAA-3’	5’-CGGCTAACGGATAAGCAGAG-3’
IFIT2	5’-ACAAGGCCATCCACCACTTTAT-3’	5’-CCCAGCAATTCAGGTGTTAACA-3’
TNF	5’-CAGAGGGCCTGTACCTCATC-3’	5’-GGAAGACCCCTCCCAGATAG-3’
HPRT	5’-GAACGTCTTGCTCGAGATGTG-3’	5’-CCAGCAGGTCAGCAAAGAATT-3’

Supplementary Figure 1. Conservation of the Arginine 841 in vertebrate WWP2 orthologues

Organism	Begin	Amino acids (green = different from human)																																													End
H.sapiens	779	F	W	Q	V	V	K	E	M	D	N	E	K	R	I	R	L	L	Q	F	V	T	G	T	C	R	L	P	V	G	G	F	A	E	L	I	G	S	N	G	P	Q	K	F	C	I	D	824
M.mulatta	779	F	W	Q	V	V	K	E	M	D	N	E	K	R	I	R	L	L	Q	F	V	T	G	T	C	R	L	P	I	G	G	F	A	E	L	I	G	S	N	G	P	Q	K	F	C	I	D	824
C.lupus	791	F	W	Q	V	V	K	E	M	D	N	E	K	R	I	R	L	L	Q	F	V	T	G	T	C	R	L	P	V	G	G	F	A	E	L	I	G	S	N	G	P	Q	K	F	C	I	D	836
B.taurus	779	F	W	Q	V	V	K	E	M	D	N	E	K	R	I	R	L	L	Q	F	V	T	G	T	C	R	L	P	V	G	G	F	T	E	L	I	G	S	N	G	P	Q	K	F	C	I	D	824
M.musculus	779	F	W	Q	V	V	K	E	M	D	N	E	K	R	I	R	L	L	Q	F	V	T	G	T	C	R	L	P	V	G	G	F	A	E	L	I	G	S	N	G	P	Q	K	F	C	I	D	824
R.norvegicus	779	F	W	Q	V	V	K	E	M	D	N	E	K	R	I	R	L	L	Q	F	V	T	G	T	C	R	L	P	V	G	G	F	A	E	L	I	G	S	N	G	P	Q	K	F	C	I	D	824
G.gallus	833	F	W	Q	V	V	K	E	M	D	N	E	K	R	I	R	L	L	Q	F	V	T	G	T	C	R	L	P	V	G	G	F	A	E	L	I	G	S	N	G	P	Q	K	F	C	I	D	878
D.rerio	775	F	W	Q	V	V	K	E	M	D	N	E	K	R	I	R	L	L	Q	F	V	T	G	T	C	R	L	P	V	G	G	F	A	E	L	I	G	S	N	G	P	Q	K	F	C	I	D	820
X.tropicalis	638	F	W	Q	V	V	K	E	M	D	N	E	K	R	I	R	L	L	Q	F	V	T	G	T	C	R	L	P	V	G	G	F	V	E	L	I	G	S	N	G	P	Q	K	F	C	I	D	683

Organism	Begin	Amino acids (green = different from human, red = R841 in human)																																													End
H.sapiens	825	K	V	G	K	E	T	W	L	P	R	S	H	T	C	F	N	R	L	D	L	P	P	Y	K	S	Y	E	Q	L	R	E	K	L	L	Y	A	I	E	E	T	E	G	F	G	Q	E	870
M.mulatta	825	K	V	G	K	E	T	W	L	P	R	S	H	T	C	F	N	R	L	D	L	P	P	Y	K	S	Y	E	Q	L	R	E	K	L	L	Y	A	I	E	E	T	E	G	F	G	Q	E	870
C.lupus	837	K	V	G	K	E	T	W	L	P	R	S	H	T	C	F	N	R	L	D	L	P	P	Y	K	S	Y	E	Q	L	R	E	K	L	L	Y	A	I	E	E	T	E	G	F	G	Q	E	882
B.taurus	825	K	V	G	K	E	T	W	L	P	R	S	H	T	C	F	N	R	L	D	L	P	P	Y	K	S	Y	E	Q	L	K	E	K	L	L	Y	A	V	E	E	T	E	G	F	G	Q	E	870
M.musculus	825	R	V	G	K	E	T	W	L	P	R	S	H	T	C	F	N	R	L	D	L	P	P	Y	K	S	Y	E	Q	L	K	E	K	L	L	Y	A	I	E	E	T	E	G	F	G	Q	E	870
R.norvegicus	825	R	V	G	K	E	T	W	L	P	R	S	H	T	C	F	N	R	L	D	L	P	P	Y	K	S	Y	E	Q	L	K	E	K	L	L	Y	A	I	E	E	T	E	G	F	G	Q	E	870
G.gallus	879	K	V	G	K	E	T	W	L	P	R	S	H	T	C	F	N	R	L	D	L	P	P	Y	K	S	Y	E	Q	L	K	E	K	L	L	Y	A	I	E	E	T	E	G	F	G	Q	E	924
D.rerio	821	K	V	G	K	E	T	W	L	P	R	S	H	T	C	F	N	R	L	D	L	P	P	Y	K	N	L	E	Q	L	R	E	K	L	L	F	A	I	E	E	T	E	G	F	G	Q	E	866
X.tropicalis	684	R	V	G	K	D	T	W	L	P	R	S	H	T	C	F	N	R	L	D	L	P	P	Y	K	S	Y	E	Q	L	K	E	K	L	L	F	A	I	E	E	T	E	G	F	G	Q	E	729

Organism	Protein Accession	Organism	Protein Accession
H.sapiens	NP_001257383.1	R.norvegicus	NP_001099654.1
M.mulatta	XP_001101869.1	G.gallus	NP_001034349.1
C.lupus	XP_851140.3	D.rerio	NP_001092918.1
B.taurus	NP_001076883.1	X.tropicalis	NP_001090841.1
M.musculus	NP_080106.1		

Supplementary Figure 2. Conservation of the Arginine 841 in 38 WWP2 paralogues

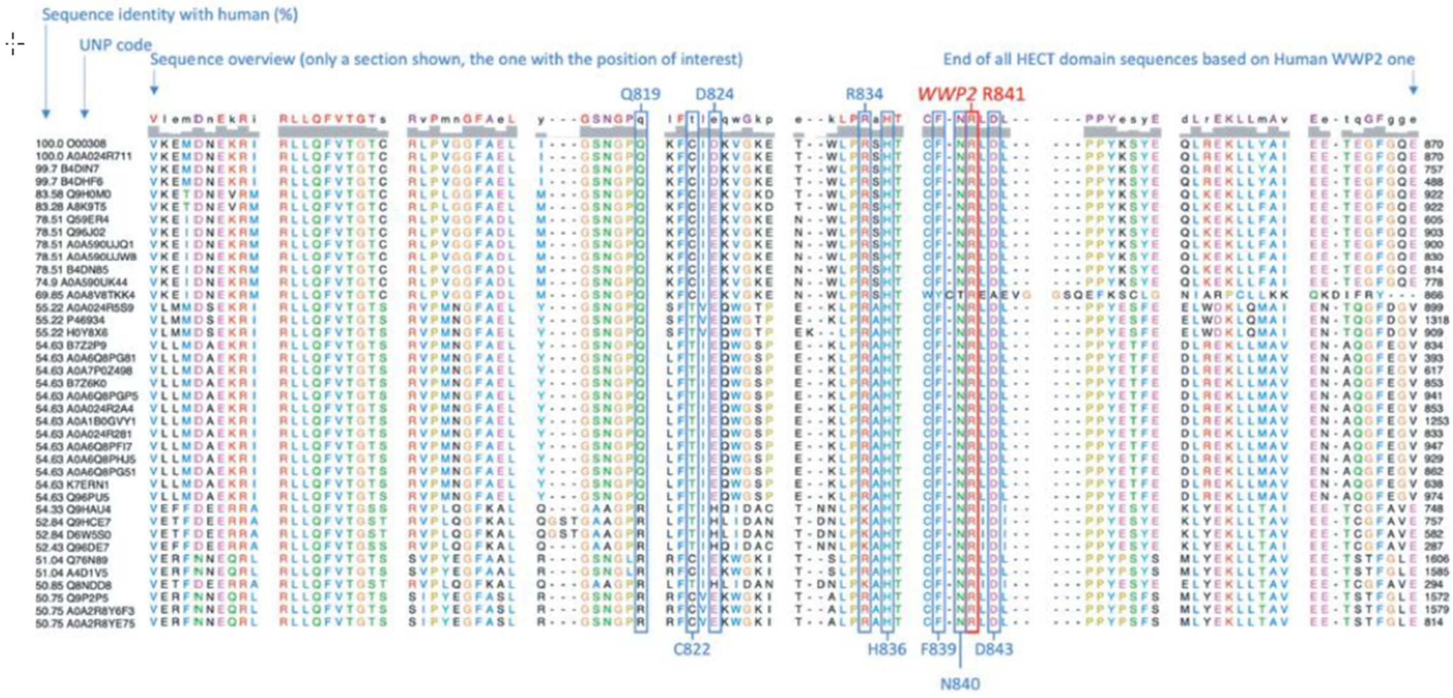

Supplementary Figure 3. Maximal and Minimal Energetic impact observed by the introduction of a R814H mutation in the HECT domain of WWP2.

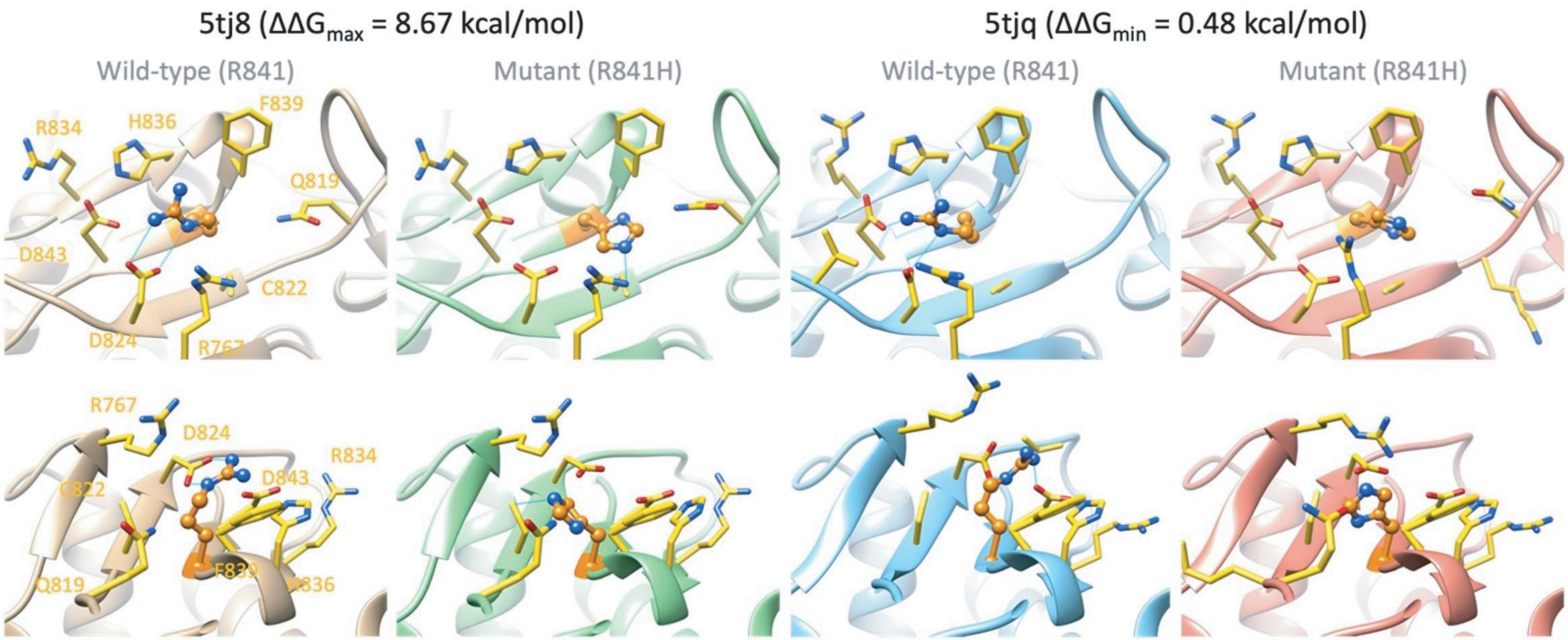

Supplementary Figure 4. WWP2-dependent IFN responses in PBMCs.

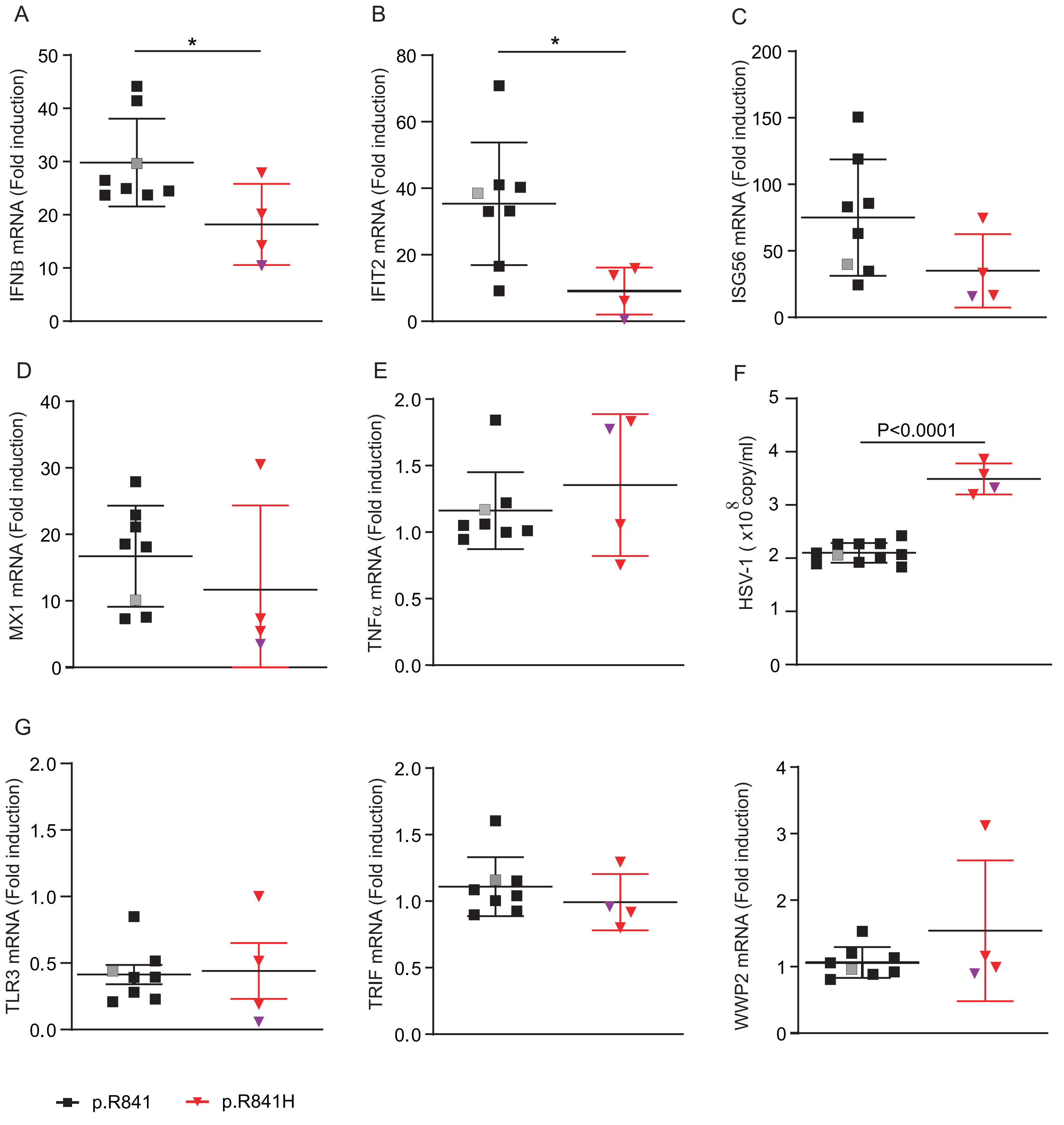

Expression of IFNβ (A), IFIT2 (B), ISG56 (C), MX1 (D), TNFα (E) as well as TLR3, TRIF, WWP2 (G) mRNA and quantification of the number of viral genome (F) in PBMCs from individuals carrying p.R841 WWP2 (black, N=8-11) or from individuals carrying p.R841H WWP2 (red, N=4) stimulated with Poly(I:C). Results represent the mean ± standard error of 1 representative experiment among 4. Statistical analyses were performed using an unpaired two-tailed Student t-test. The purple triangle and the grey square correspond to patient with HSE and to patient's father respectively.
* indicates P=0.0405(A) and P=0.02 (B).

Supplementary Figure 5 Significant downregulation of NFKB pathway downstream target genes in WWP2 p.R841H compared to WWP2 p.R841.

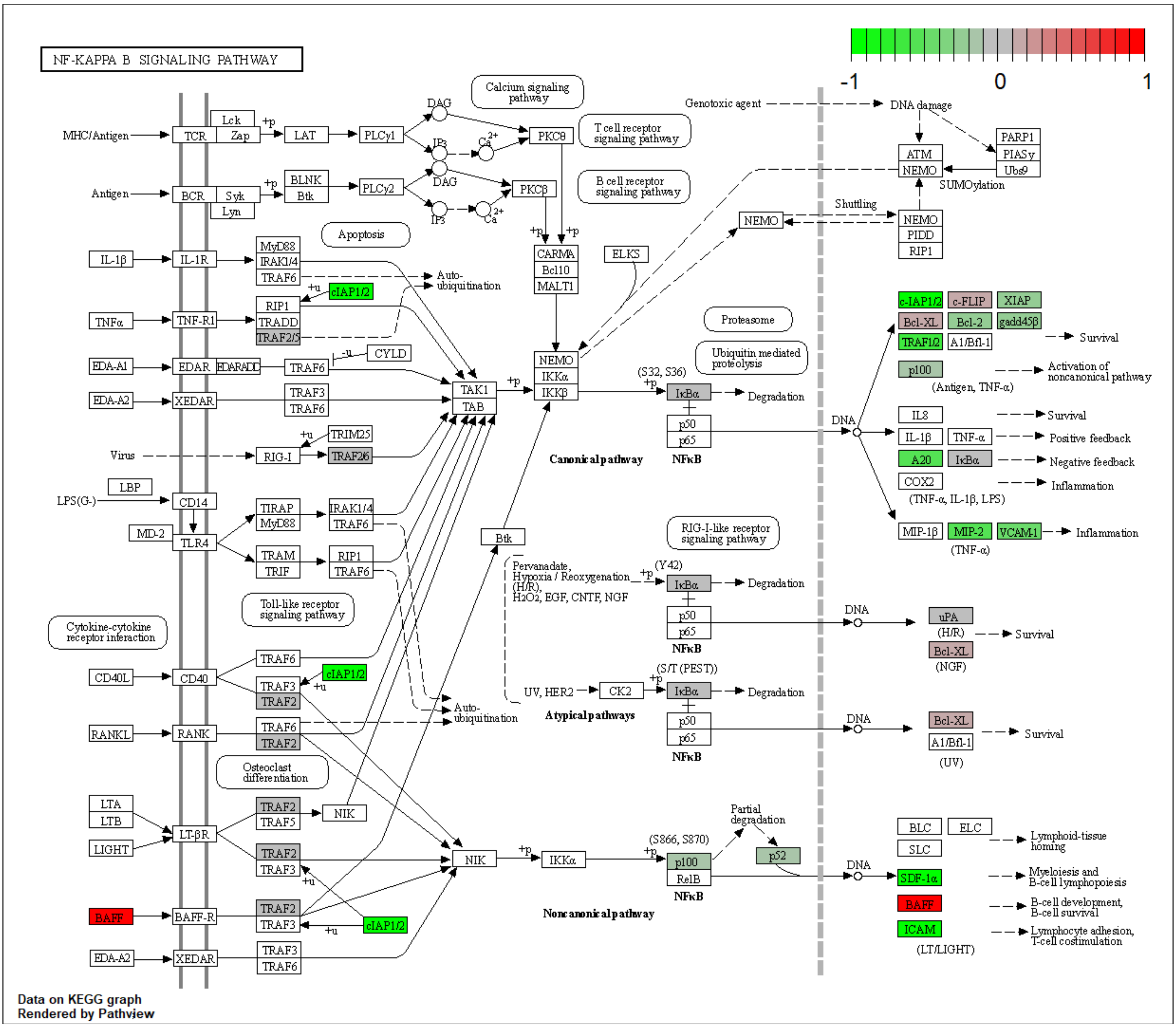

Supplementary Figure 6. Significant downregulation of AP-1 downstream target genes in WWP2 p.R841H compared to WWP2 p.R841.

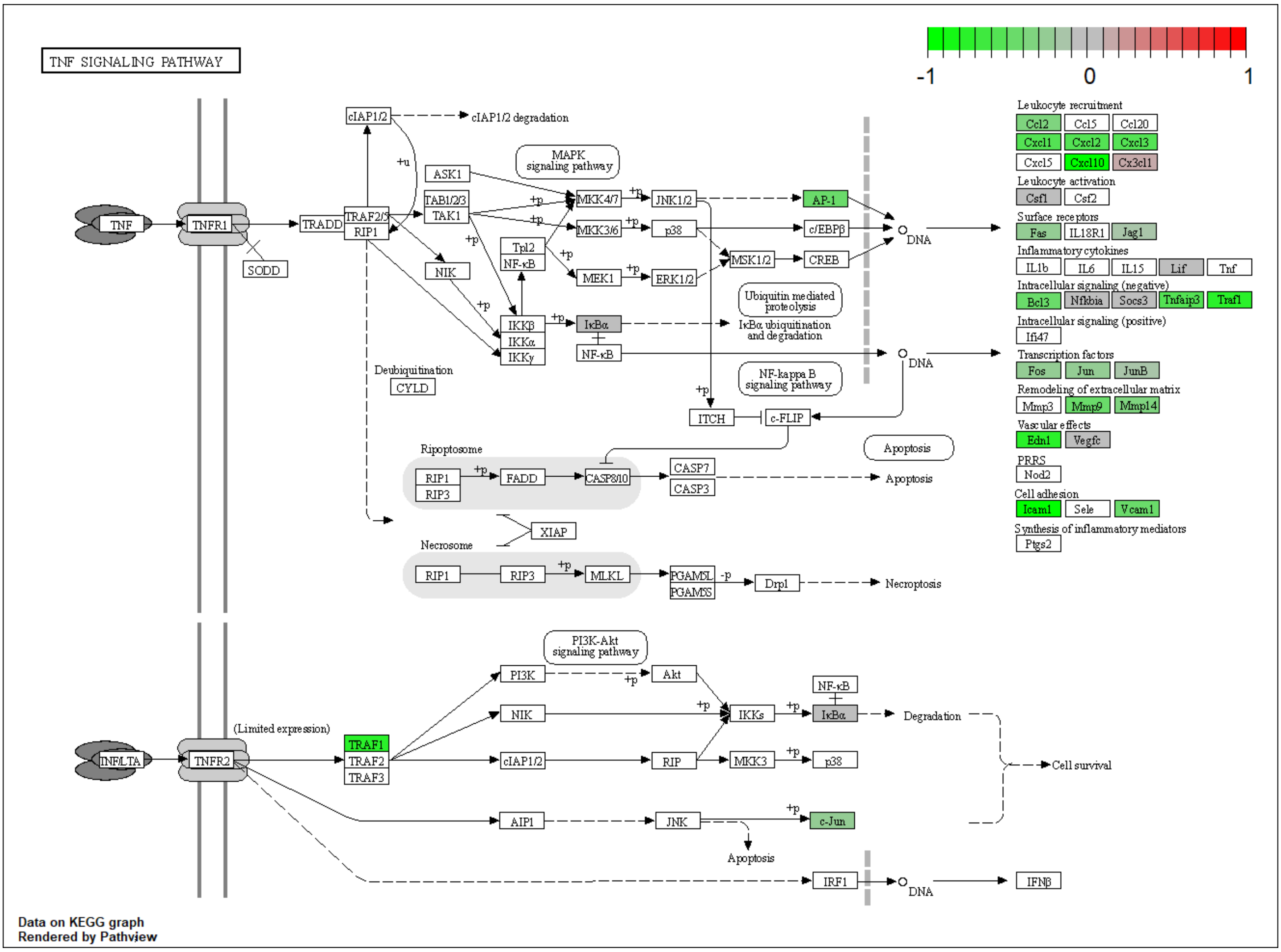

Supplement: Supplementary file 1 — Supplementary Information [file 41467_2024_48287_MOESM1_ESM.pdf]
